# Low Frequency Vibrations Disrupt Left-Right Patterning in the Xenopus Embryo

**DOI:** 10.1371/journal.pone.0023306

**Published:** 2011-08-03

**Authors:** Laura N. Vandenberg, Brian W. Pennarola, Michael Levin

**Affiliations:** 1 Center for Regenerative and Developmental Biology, Tufts University, Medford, Massachusetts, United States of America; 2 Biology Department, Tufts University, Medford, Massachusetts, United States of America; Radboud University Nijmegen, The Netherlands

## Abstract

The development of consistent left-right (LR) asymmetry across phyla is a fascinating question in biology. While many pharmacological and molecular approaches have been used to explore molecular mechanisms, it has proven difficult to exert precise temporal control over functional perturbations. Here, we took advantage of acoustical vibration to disrupt LR patterning in *Xenopus* embryos during tightly-circumscribed periods of development. Exposure to several low frequencies induced specific randomization of three internal organs (heterotaxia). Investigating one frequency (7 Hz), we found two discrete periods of sensitivity to vibration; during the first period, vibration affected the same LR pathway as nocodazole, while during the second period, vibration affected the integrity of the epithelial barrier; both are required for normal LR patterning. Our results indicate that low frequency vibrations disrupt two steps in the early LR pathway: the orientation of the LR axis with the other two axes, and the amplification/restriction of downstream LR signals to asymmetric organs.

## Introduction

In vertebrates, external bilateral symmetry with respect to the left-right (LR) axis is coupled with a variety of internal asymmetries that are consistently biased in all normal individuals [1], [2]. For example, conserved LR asymmetry of the internal organs includes the shape and placement of the heart, liver, gall bladder, stomach and lungs. Moreover, abnormalities in laterality form a class of human birth defects that can seriously impact the health of affected individuals [3], [4], [5]. These defects can be classified into several types: *situs inversus*, the complete inversion of all body organs resulting in mirror-image organ position compared to normal individuals; *isomerism*, where the individual develops symmetrically, resulting in organs that are duplicated or missing completely; *dextrocardia* (where the heart is located on the wrong side) and other single organ inversions; and *heterotaxia*, a loss of concordance in which the location of each organ is determined independently from all other organs. The consistent asymmetry between the left and right sides of many organisms proposes a puzzling developmental problem. How are all embryos able to pattern the LR axis consistently despite a bilaterally symmetrical environment?

Two main models have been proposed to explain consistent embryonic laterality. The popular nodal flow model proposes that the inherently clockwise rotation of cilia located on cells isolated in a small pocket or node of the late gastrula- and early neurula-stage embryo creates a leftward fluid flow [6], [7], [8], [9], [10], [11], [12]. This asymmetric flow is transduced into asymmetric gene expression by direct redistribution of morphogens and/or the activation of sensory cilia on one side of the node [9], [13], [14]. However, a number of facts have suggested the need for alternate models that focus on much earlier events occurring prior to gastrulation [15], [16], [17], [18], [19].

Animals in many diverse phyla set up the LR axis without cilia, including snails [20], [21], sea urchins [22], *Drosophila* [23], [24], *Arabidopsis* [25], [26], [27], *C. elegans* [28], [29] and pigs [30]. Furthermore, many model organisms establish their LR asymmetry prior to gastrulation, stressing the importance of earlier mechanisms, which is the focus of the second model of embryonic laterality. The cytoskeleton of the vertebrate embryo has a LR-biased chirality, which is present even prior to the first cell cleavage [31] and is likely an ancient property of all cells [32]. Within the first embryonic cell divisions, the biased cytoskeleton drives differential localization of maternal protein cargo along the LR axis [33], [34] including two potassium channels [33], [35] and two proton pumps [36], [37]. Because the cells on the right side of the embryo are more negative (due to the release of K^+^ and H^+^ ions), electrical gradients, together with a network of open gap junctions, allow LR signaling molecules such as serotonin to be distributed to the right- and ventral-most blastomere [38], [39]. To protect and maintain the voltage gradient, tight junctions must preserve the integrity of the epithelium that participates in this multicellular electric circuit. Alterations to tight junctions, therefore, result in significant disruptions of the LR axis [40], [41]. Together, all of these steps are required for LR orientation and are upstream of asymmetric gene expression and organ *situs* [42]. While most of these steps have been characterized in detail in the Xenopus model, similar roles for ion flux [20], [43], [44], [45], [46], gap junctions [47], serotonin [39], and tight junctions [48], [49] have been observed in a variety of other species including *Arabidopsis*, *C. elegans*, sea urchins, snails, rabbits, and chicks, among others.

The functional investigation of asymmetry mechanisms requires the ability to perturb intracellular structures and cell∶cell interactions at precise stages of development. However, molecular-genetic alterations do not offer temporal control (especially for maternal mRNA or protein targets, where inducible promoters cannot be used). For example, genetic mutations [6], [8] exert effects throughout development; likewise, once microinjected, mRNAs cover very broad segments of embryogenesis [36], [41], [50]. Pharmacological approaches [51], while offering some degree of temporal control [38], [39], are not ideal because of issues including unknown half-life and metabolism, (in)accessibility of internal structures, and concern over when the chemical is truly “washed in or out” of the system [52].

Thus, we sought a treatment that would avoid problems of penetration into deep tissues, and most importantly, would afford precisely-delimited ON and OFF states for perturbation of embryonic events. A few studies (mostly *in vitro*) have examined the effects of physical vibrations. Vibration protocols that were designed to mimic the intensity and duration of vibrations produced during the launch phase of space flights altered the expression of several cytoskeletal genes in Jurkat T lymphocytes including cytoplasmic actin, α-tubulin, keratin, and C-NAP1, a centriole-organizing protein [53]. The microtubule network and the microtubule organizing centers (MTOCs) were also disorganized. Similar results have been obtained for other cell types including breast cancer cells and whole sea urchin embryos [54]. Thus, vibrating embryos provides a promising method by which the cytoskeleton and other aspects of embryonic organization can be targeted in *Xenopus* embryos. Because vibration can be initiated and stopped at exact timepoints, and because of the several studies implicating cytoskeletal dynamics in LR asymmetry in a wide range of phyla including vertebrates [23], [27], [28], [31], [33], [34], [55], [56], acoustic vibration was used to explore novel aspects of LR patterning in frog embryos.

We hypothesized that physical perturbations via low frequency vibrations would disrupt the orientation of the LR-axis in a period-specific manner. We expected that vibrations would be most effective during the first few cleavage stages, during the period when the chiral cytoskeleton plays a critical role in orienting the LR axis relative to the anterior-posterior and dorsal-ventral axes [33], and is required for asymmetric localization of ion transporters and other proteins [31], [33], [34], [35], [37]. Here, we identify several low frequency vibrations that specifically randomize the LR axis, and explore the effects of one, 7 Hz, at different stages of development. While we confirm a strong effect of vibration during the first cell cleavage, we also identify a second period of sensitivity to vibrations and show that treatment during this sensitive period disrupts tight junctions, an important part of the LR pathway.

## Results

### Low frequency vibrations alter orientation of the LR axis

**Figure 1:**
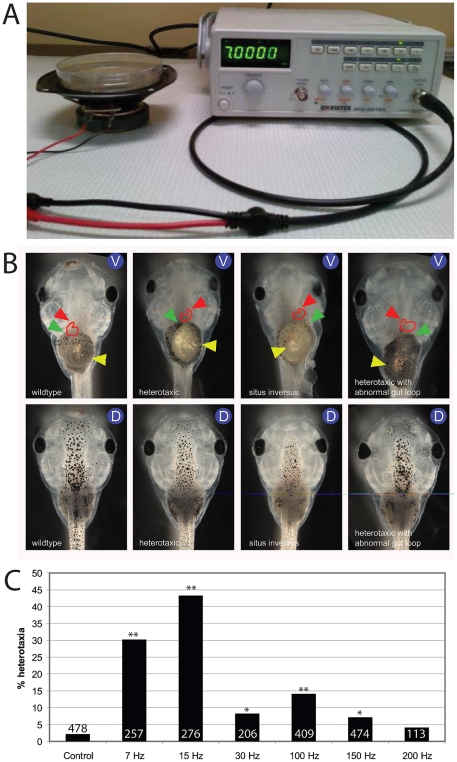
Heterotaxia is induced by various low frequency vibrations. A) A simple experimental device was produced from a Gwinstek GFG-8216A function generator (right) attached to a 4-inch Sony speaker (left). A petri dish, with the appropriate solution containing embryos, was placed on top of the speaker at various stages. B) Ventral views (V) showing organ position and dorsal views (D) showing normal dorsoanterior development in tadpoles with wildtype situs, heterotaxia, and situs inversus. In all panels of ventral views, the red arrowhead indicates the apex of the heart, yellow indicates the stomach, and green indicates the gall bladder. For wildtype organ situs, the apex of the heart loop is located on the animal's right side, the stomach coils to the animal's left, and the gall bladder is positioned on the animal's right. The heterotaxic tadpole shown here has an inverted heart, but normal position of the stomach and gall bladder. There are seven combinations of position of the three scored organs that are each examples of heterotaxia, including *situs inversus*, where all three organs are inverted. The right-most panel also shows an animal with heterotaxia, with an inverted stomach and gut, but normal position of the heart. Note that the coiling of the stomach is also abnormal, a phenotype observed in a minority of vibrated embryos (<5% at 7 Hz). C) Quantification of heterotaxia in embryos vibrated at different frequencies from 1 cell through st. 19 of development. 7 Hz and 15 Hz were the most effective at randomizing the LR axis, but 15 Hz vibration also produced other developmental abnormalities in a large subset of embryos (not shown); therefore, 7 Hz was selected for additional study. Numbers on bar graphs indicate sample sizes from a combination of two or more replicate studies. * p<0.01, but not biologically relevant because heterotaxia rates were below 10%, the minimal meaningful difference. ** p<0.001, χ^2^ test.

Previous studies have shown that pharmacological reagents that affect different components of the cytoskeleton can induce laterality defects in *Xenopus* embryos [34], [37], even when the cytoskeleton is targeted prior to the first cleavage [31]. In other systems, particularly *in vitro* culture of human cells, physical vibrations have been linked to alterations in the cytoskeleton [54]. To address whether vibration could affect LR asymmetry, we used a speaker driven by a digital signal generator to vibrate embryos (covering the range of 7 Hz to 200 Hz) from the 1 cell stage through neurulation (typically to stage 19, an overnight period) (Figure 1A). Scoring the position of the heart, stomach, and gall bladder at stage 45, we observed that several of these frequencies specifically induced heterotaxia (an independent assortment of individual organ inversions) in a significant proportion of exposed embryos (Figure 1B–C). Low frequencies were the most effective of the vibrations we tested. Inversion of the stomach was determined only by the direction of gut looping, but we also noted that other defects in gut coiling were present in some treated embryos (less than 5% after vibration at 7 Hz, see Figure 1B, far right panel); we did not characterize these defects. While we only scored asymmetry in embryos with otherwise normal development (Figure 1B, see dorsal images), vibration at 15 Hz also induced neural tube defects and was significantly more toxic than the other frequencies examined (data not shown); thus, for the remainder of our analyses, we focused our efforts on 7 Hz vibration because it was effective at disrupting the LR axis but produced few other defects.

Because speakers produce electromagnetic fields (EMFs) in addition to physical vibrations, we wanted to determine whether EMFs alone could be responsible for the effects we observed [57], [58], [59], [60]. Using a speaker with the stationary magnet removed (allowing for the exact same EMFs to be produced in the absence of physical vibration), we exposed embryos from 1 cell to st. 19, or several other developmental periods. None of these EMF exposures had an effect on the LR axis (Figure S1). We conclude that EMFs do not affect the orientation of the LR axis, and that a sinusoidal signal-driven speaker system is an effective and inexpensive method for specifically disrupting asymmetry via mechanical perturbation.

### Stage-specific vibration exposure implicates two periods of sensitivity

**Figure 2:**
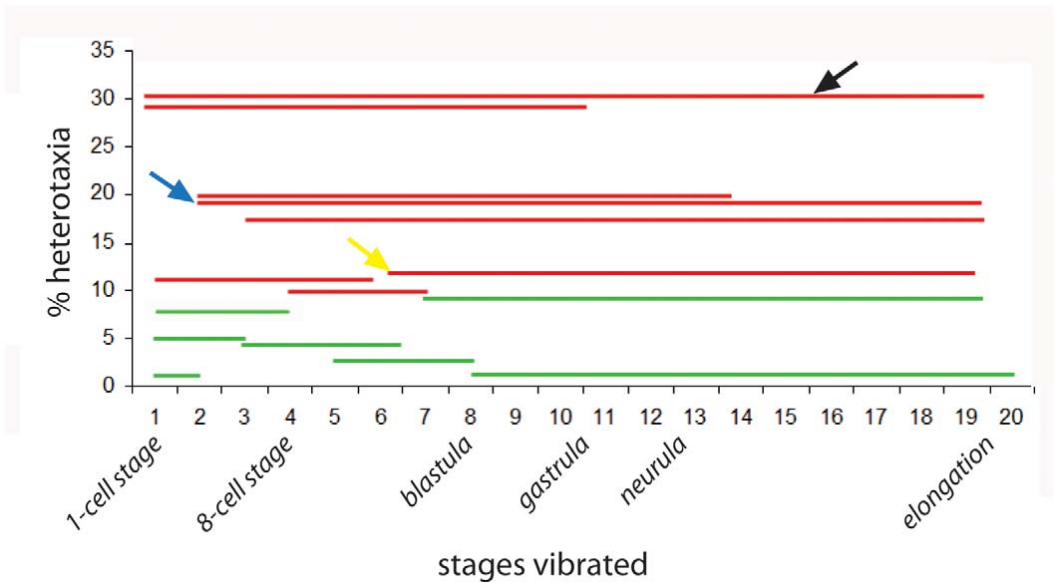
Vibration period influences the frequency of laterality defects. When embryos were vibrated at 7 Hz during different periods of development, striking differences in the rate of heterotaxia were observed. Most effective were vibrations that started at 1 cell and went through mid-gastrula (st. 10.5) or neurula stages (st. 19, see black arrow). Somewhat less effective were vibrations starting just a short time later, at the 2 cell stage (blue arrow). Finally, vibrations that included a second period of time, around st. 6, were able to disrupt left-right patterning, but to a lesser degree (see yellow arrow, for example). For all groups, a red line indicates significant differences from non-vibrated controls (χ^2^ test, p<0.01) and heterotaxia rates above the minimal meaningful difference of 10%. A green line indicates that the treatment either was not significantly different from controls or was below the minimal meaningful difference of 10% heterotaxia. All lines are the summed data from two or more replicate studies.

To identify specific stages at which developmental mechanisms might be sensitive to mechanical disruption of LR patterning, we vibrated embryos at 7 Hz for different periods of time, starting and ending at different developmental stages. We observed that vibration from 1 cell to st. 19 was most effective at disrupting the LR axis (Figure 2, black arrow). Strikingly, initiating vibration just 60 minutes later (at the 2-cell stage) reduced the incidence of heterotaxia by approximately 33% (Figure 2, blue arrow). This result suggests that the period encompassing the first cleavage is especially sensitive to vibration. Importantly, vibration from the 1 cell to 2 cell stage alone did not randomize LR patterning, suggesting that vibration during this period is necessary, but not sufficient, for maximum disruption of the LR axis.

Vibrations for various other durations revealed a second possible period that was sensitive to vibration: the window between st. 4 (8 cell) and st. 7. Short vibration periods that encompassed this entire developmental window induced heterotaxia in approximately 10% of embryos, whereas other short vibration periods that only covered portions of this window were less effective (Figure 2). Further implicating this second sensitive period, vibrations from st. 6 through neurulation induced heterotaxia in approximately 12% of embryos (Figure 2, yellow arrow), but vibrations starting just a few hours later at st. 8, also continuing through neurulation, had no significant effects on the LR axis. While the penetrance of heterotaxia was relatively low after these later vibrations, similar rates of laterality problems have been reported for well-established LR treatments (see for example mutants and molecular manipulations reported in [61], [62], [63]). From these results, we conclude that a period around stages 4–7 is sensitive to vibration, but to a lesser degree than vibrations starting earlier in development.

### Vibrations during two discrete periods increase the incidence of laterality defects

**Figure 3:**
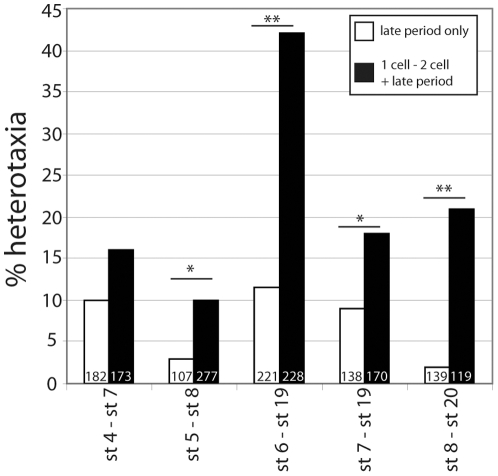
Vibration from 1 cell to 2 cell, paired with various later vibration periods, increases the rate of heterotaxia. One set of embryos was vibrated for a select period of time later in development (white bars). A second set was vibrated from 1 cell to 2 cell, allowed to rest in a vibration-free environment, and then re-vibrated for the same select period of time later in development (black bars). In all cases, adding vibrations from 1 cell to 2 cell increased the incidence of heterotaxia observed at st. 45. Vibrations from 1 cell to 2 cell without a later period of vibration were not significantly different from untreated controls (not shown here, see Figure 2). For statistical comparison, the actual numbers of embryos from two or more replicate studies with wildtype versus heterotaxic situs were compared for single late vibrations and the two paired vibrations (χ^2^ test, *p<0.05, **p<0.001).

Our results clearly implicated an effect of vibration during the first cell cycle; groups of embryos vibrated from 1 cell through neurula stages were more affected than those vibrated from the 2 cell stage (Figure 2). However, vibration from the 1 cell to 2 cell stage was not by itself sufficient to alter LR patterning. To determine if this early period of vibration would have additive effects on a second discrete period of vibration, we vibrated embryos from 1 cell to 2 cell, removed them from vibration, and then resumed vibration at later periods for various developmental windows. For many vibration periods, adding an early vibration during the first cell cleavage enhanced the effect of later vibrations (Figure 3). The most striking example was observed in embryos vibrated from st. 6 through neurulation; embryos vibrated during this period alone had a rate of 11.5% heterotaxia. Adding in an early vibration from 1 cell to 2 cell increased the incidence of heterotaxia obtained from this treatment to 42%. From these results, we conclude that there are two main periods of sensitivity to low frequency vibrations, and that treatments including both periods of sensitivity have additive effects.

### Vibration affects LR-relevant events upstream of asymmetric gene expression

**Figure 4:**
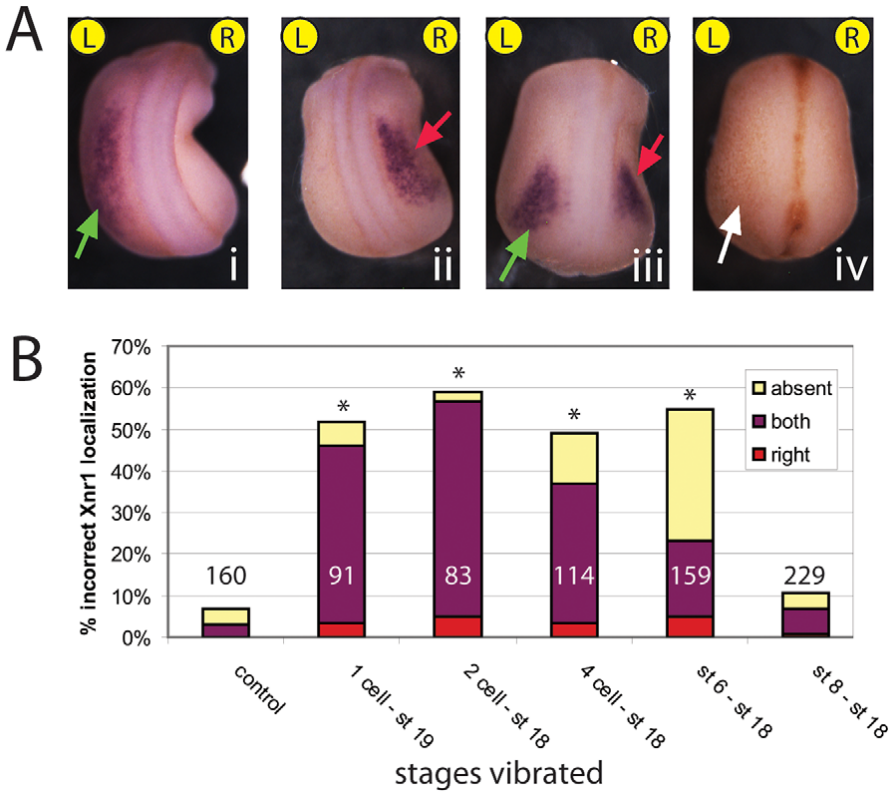
Localization of the asymmetric gene Xnr-1 is affected by vibration during early, but not late, cleavage stages. A) Control and vibrated embryos were examined at approximately st. 22 for the localization of Xnr-1 mRNA. All embryos were classified as having correct, left-sided expression (i), or incorrect right-sided (ii), bilateral (iii) or absent (iv) expression. Green arrows indicate correct expression, red indicate incorrect expression, and white indicate absent expression. All embryos are positioned with the anterior upward and the left (L) and right (R) sides are indicated. B) Quantification of incorrect Xnr-1 localization in control and vibrated embryos. When vibrations started at 1 cell, 2 cell, 4 cell or st. 6, there was a significant increase in incorrect Xnr-1 expression compared to controls (χ^2^ test, *p<<0.001). Vibrations starting at st. 8 were not statistically different from untreated controls.

In Xenopus and other model species, there are well-characterized genes with asymmetric expression that are upstream of the asymmetric morphogenesis and position of the heart and visceral organs. We next asked whether vibrations disrupted the most widely conserved asymmetric gene, Nodal (Xnr-1). Xnr-1 is normally expressed only on the left side of the embryo at approximately stage 20 [64]. Vibrations starting at 1 cell, 2 cell, 4 cell or st. 6 and continuing through neurulation all significantly disrupted Xnr-1 localization (Figure 4). Mislocalization of Xnr-1 was observed in 49–59% of the vibrated embryos, depending on the period of treatment. Vibration from st. 8 through neurulation did not significantly affect Xnr-1 localization (Figure 4); consistently, this same treatment also did not significantly affect organ situs. From these results, we conclude that the LR orientation mechanisms disrupted by low frequency vibrations feed into the well-conserved transcriptional cascades regulating organ situs [65], [66], and function upstream of asymmetric Xnr-1 expression.

### Different periods of vibration produce variable “signatures” of inverted organs

**Figure 5:**
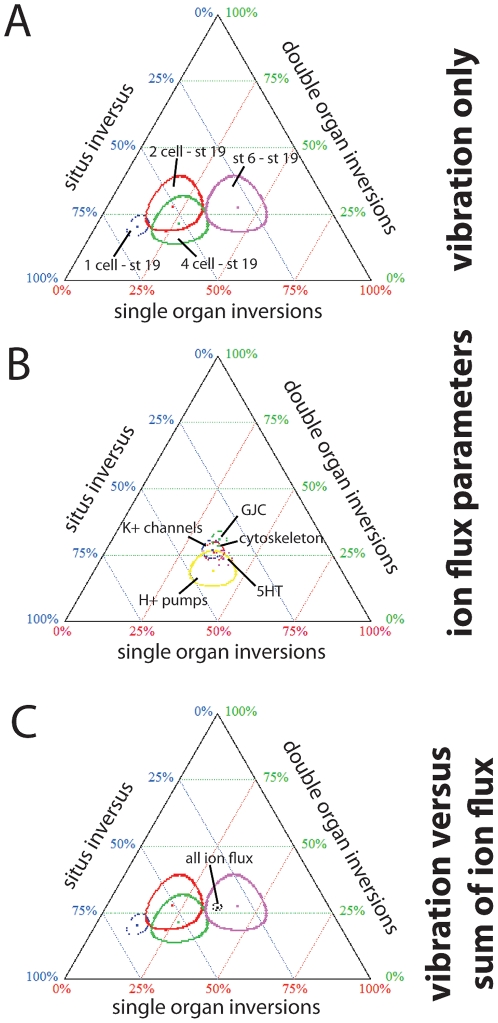
Ternary plots reveal unique signatures of inverted organs depending on the timing of vibration treatment. All heterotaxic embryos were compiled by treatment and graphed on a ternary plot where one axis represents the % of embryos with single organ inversions, the second axis represents the % of embryos with two (double) organ inversions, and the third axis represent the % of embryos with *situs inversus* (all three organs inverted.) Data is graphed so a single point represents each treatment, surrounded by an oval that illustrates the 95% confidence interval (CI); when the ovals do not overlap, the groups are significantly different from each other [67]. A) Unique signatures of embryos vibrated from 1 cell to st. 19 (blue), 2 cell to st. 19 (red), 4 cell to st. 19 (green) or st. 6 to st. 19 (violet) demonstrate that the period of vibration significantly affects the organ inversions observed. Most striking are the differences in the incidence of situs inversus, which is highest in embryos vibrated from 1 cell to st. 19 and lowest in embryos vibrated from st. 6 to st. 19. B) Signatures of affected organs are very similar in heterotaxic embryos from groups treated with chemicals and molecular constructs targeting H^+^ pumps (yellow), K^+^ channels (blue), gap junctional communication (GJC, green), the cytoskeleton (red), and the LR signaling molecule serotonin (5HT, purple). For a complete list of the reagents used, see Supplemental Table 2. C) The ion flux data shown in panel B was combined and statistically collapsed to produce a datapoint that represents all ion flux-related treatments (black dots, inside the violet circle). This was compared to the signatures obtained from the vibration protocols (panel A). The ion flux data thus overlaps with the signatures obtained from vibrations occurring from st. 6 through neurulation (violet).

We next analyzed the detailed distribution of different combinations of the three organ positions among each treatment (7 possible outcomes in all). We observed that embryos exposed to low frequency vibrations from 1 cell to st. 19 had a large proportion of the affected embryos developing situs inversus rather than discordant heterotaxia (Figure 1B). If each of the three organs independently assumes a right or left position in a truly random manner, each combination of the 7 possible organ distributions is expected to occur 14.3% of the time. To determine whether the timing of vibration exposure affected the distribution of heterotaxia phenotypes, we graphed the affected embryos on a ternary plot [67], where one axis defined the % of affected embryos with single organ inversions (expected value for random placement = 14.3%×3 = 42.9%), the second axis defined the % of affected embryos with double organ inversions (expected value for random placement = 14.3%×3 = 42.9%), and the third axis defined the % of affected embryos with all organs inverted (situs inversus, expected value = 14.3%). Using this graph, we observed that distinct windows during which embryos were vibrated produced unique “signatures” on the ternary plots. Embryos vibrated from 1 cell to st. 19 had a higher percentage of affected embryos with situs inversus (Figure 5A, blue), whereas embryos vibrated from 2 cell or 4 cell through neurulation had significantly less situs inversus and more single organ inversions (Figure 5A, red). Contrasting further, embryos vibrated from st. 6 through st. 19 had even fewer affected embryos with situs inversus, and more with single organ inversions (Figure 5A, violet). In all 4 vibration groups examined, there was relatively little change in the percentage of affected embryos with double organ inversions. From these results, we conclude that the timing of vibration significantly and specifically affects the signature of affected organs, with the greatest differences due to changes in the ratios of heterotaxic embryos with one versus three organ inversions.

In order to compare these signatures to the outcomes of other, well-characterized pathway perturbations, we analyzed the primary data of published studies in which the position of all three organs was reported following treatment with a molecular or pharmacological reagent to disrupt orientation of the LR axis [34], [37], [38], [39], [68], [69], [70], [71]. Comparing the % of affected embryos with single or double organ inversions and situs inversus from embryos treated with chemicals or molecular constructs targeting the cytoskeleton, H^+^ pumps, K^+^ channels, gap junctional communication, and serotonergic signaling (see Figure S2 for a complete list of reagents), we observed that the inverted organ signatures obtained from these reagents were very similar (Figure 5B).

The vibration periods that are most effective at randomizing the LR axis overlap with the early developmental stages implicated in the ion flux pathway (including the early cytoskeleton, H^+^ pumps, K^+^ channels, gap junctions and serotonin) [42], [72], [73], [74]. In order to determine whether vibrations during different developmental windows produced similar affected organ signatures as the reagents targeting specific aspects of the ion flux pathway, we compared several vibration treatments with the signature obtained from the sum of all ion flux data. We observed that vibrations from 1 cell to st. 19 were the most different from treatments targeting ion flux, and as the period of vibration started later in development, they became more similar to treatments targeting ion flux (Figure 5C). Ultimately, vibrations starting at st. 6 and continuing through neurulation were not statistically distinguishable from chemicals that target ion flux, suggesting that this later period of sensitivity may be related to mechanisms implicated in the ion flux LR pathway.

From these data, we conclude that the “signature” of inverted organs obtained from vibration of embryos is unique to the developmental window when vibration occurred. Vibrations starting at 1 cell are clearly different from other treatments – including vibrations that started later in development and chemicals targeting the ion flux pathway – because they induce large amounts of situs inversus. Because individuals with situs inversus maintain LR asymmetry but orient their LR axes randomly compared to the other two axes, these results may suggest that early vibrations uncouple a mechanism that is needed to orient the LR axis with the other axes; in contrast, later vibrations disrupt the concordance of organs similar to reagents that affect the ion flux pathway.

### Vibration affects nocodazole-sensitive LR pathways

**Figure 6:**
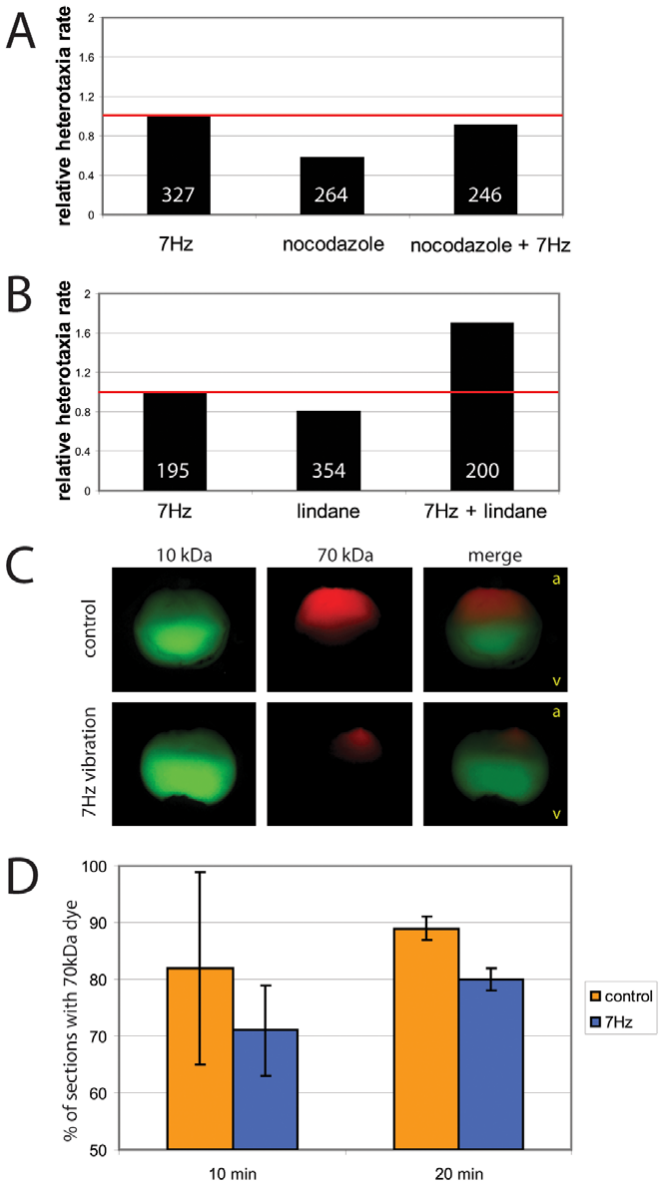
Epistasis and dye movement experiments indicate that low frequency vibrations alter nocodazole-sensitive pathways and intracellular movements. For epistasis experiments, embryos were vibrated, treated with a drug, or given a combination of these two treatments from 1 cell to st. 12, and organ laterality was assessed at st. 45. If the combination produced an additive effect, we concluded that these treatments were likely affecting different aspects of the left-right (LR) pathway; if the combination was not additive, we concluded that the treatments were likely targeting the same aspects of the LR pathway. A) Embryos were vibrated, treated with the microtubule disruptor nocodazole, or a combination of the two. No additive effects on heterotaxia rates were observed, therefore it is likely that vibrations target nocodazole-sensitive LR pathways. B) In contrast, embryos treated with lindane, a gap junction blocker, and vibration showed additive effects over each treatment alone. These results show that additive effects can be obtained, and indicate that vibrations are not likely to be targeting gap junctions. C) 1 cell embryos were injected in the central area of the animal pole with a mixture of two dyes, then vibrated or left untreated and examined 10 minutes later. In both treatments, the low molecular weight dye (10 kDa) spread throughout most of the embryo with the strongest concentration in the vegetal hemisphere. In controls, the high molecular weight dye (70 kDa) typically spread throughout the top third of the embryo, whereas the same dye remained localized much more strongly to a single point in the upper quadrant of animal pole in the vibrated embryo. Orientation of the embryos is indicated in the merged panels with a (animal) and v (vegetal). D) Sections were obtained from 1 cell embryos after 10 or 20 minutes of vibration, and time-matched unvibrated controls. The percentage of A/V sections containing TMR dye were calculated, giving a quantitative measure of how far the dye had spead laterally through the embryo. The data indicate that over time, the high molecular weight dye spread out laterally in control embryos, but in vibrated embryos, the dye spread to a lesser degree.

Previous studies have implicated the cytoskeleton in early asymmetries apparent in Xenopus embryos, and functional studies have shown that early alterations in either microtubules or the actin cytoskeleton can induce heterotaxia [31], [33], [34]. Because vibration has been shown to affect the cytoskeleton of cells [53], [54], we first performed epistasis experiments to determine whether vibration was affecting the same pathway as nocodazole, which interferes with microtubule polymerization. Embryos that were treated with both nocodazole and also vibrated from 1 cell to stage 12 had no overall increase in the incidence of heterotaxia (were not additive) (Figure 6A). From these results, we conclude that vibration is likely affecting the same LR pathway components as does the cytoskeletal drug nocodazole, consistent with the effect of vibration on cytoskeletal organization *in vitro*.

We also performed epistasis experiments to determine whether vibrations could affect another target, gap junctional communication, using lindane, a gap junction blocker [75]. Gap junctions are an essential component of the LR pathway in several organisms including Xenopus, chick, rabbit and *C. elegans* [46], [47], [75], [76]. We found that in contrast to our experiments with nocodazole, co-treatment with lindane and vibration from 1 cell to stage 12 produced additive effects with almost double the number of heterotaxic embryos compared to vibration or lindane exposure alone (Figure 6B). These results suggest that vibration is not affecting gap junctional communication in general, or is not targeting gap junctions in the same manner as the general gap junction inhibitor lindane.

### Vibration affects distribution of molecules within the embryonic cytoplasm

In the early cleavage stage Xenopus embryo, the cytoskeleton is required for the asymmetric movement of LR-relevant cargo throughout the cytoplasm [33], [34], [35], [37], similar to the role of the cytoskeleton in the transport of dorsal determinants during specification of the dorsal-ventral axis [77] and the movement of cargo along cytoskeletal tracts in differentiated cells [78]. To determine whether low frequency vibrations affect the movement of molecules within the embryo, we injected embryos in the centermost point of the animal pole with a mixture of two fluorescent dyes with different molecular weights, and then compared the distribution of the dyes in vibrated versus control embryos that were sectioned along the animal-vegetal plane. We observed that the low molecular weight dye (10 kDa) distributed throughout the embryos, with no qualitative differences between treatments. In contrast, we observed striking differences in the distribution of high molecular weight dye (70 kDa) between vibrated and control embryos (Figure 6C). The 70 kDa dye diffused deeper into the control embryo, whereas in the vibrated embryo the dye remained localized to a smaller “node” in the animal hemisphere. Furthermore, when we examined the number of 100 µm cross-sections that contained the high molecular weight dye, we observed that the dye spread outward (laterally, into more individual sections) in the control embryos to a higher degree than was observed in the vibrated embryos (Figure 6D). These results together suggest that vibration affects the cytoplasmic movement of large molecular weight molecules in both the animal-vegetal axis as well as the lateral direction of the 1 cell embryo.

### Vibration disrupts integrity of the epithelium in st. 6 Xenopus embryos

**Figure 7:**
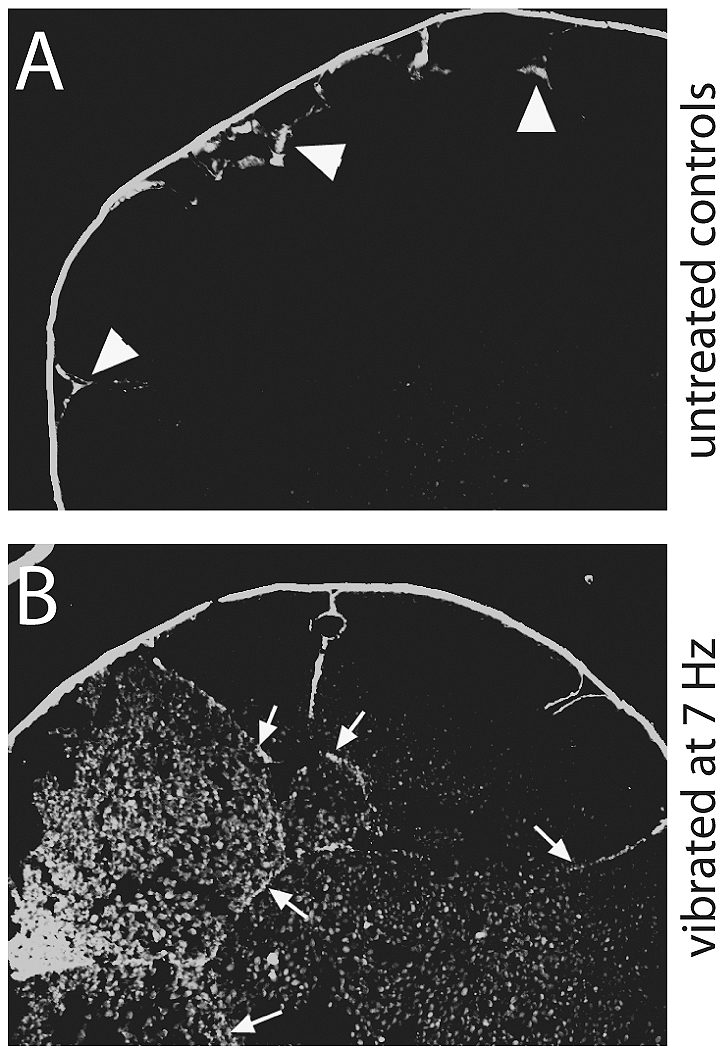
Epithelial integrity is disrupted in vibrated embryos. A) Using a biotin permeability assay, we determined that epithelial integrity was maintained in control embryos such that the biotin signal was normally observed at the outer edges of the embryo, less than 1 cell depth (arrowheads). B) In vibrated embryos, the biotin signal was observed much farther into the embryo, with signal several cell layers deep (arrows).

Our initial examination of the effects of vibration during different developmental periods implicated a second period of sensitivity to vibration around st. 6 (Figure 2). Previous studies indicate that this is a period where tight junction integrity is essential for LR asymmetry [40], [41]. To determine whether vibration disrupts integrity of the normally impermeable epithelial barrier, we performed a biotin permeability assay on vibrated and control embryos. In this assay, a biotin molecule that was modified to have long spacer arms and a charged sulfo-group to prevent it from passing through normal tight junctions was used. This molecule covalently bonds with primary amine groups on the external surfaces of the cell unless tight junctions between cells are compromised [40]. In control embryos at st. 6, we observed that biotin was, as expected from the known pattern of tight junctions [79], typically localized only to the surface of the embryo, with penetration limited to less than half the depth of the outer cell layer (Figure 7). In contrast, embryos vibrated from 1 cell to st. 6 often showed localization of biotin several cell layers deep at st. 6 (Figure 7). These results are consistent with a disruptive effect of vibration on epithelial integrity.

## Discussion

Here, we show for the first time that physical perturbations induced by low frequency vibrations are sufficient to alter orientation of the LR axis in a vertebrate animal. While we observed that several frequencies disrupted LR patterning, we focused our efforts on examining the effects of 7 Hz, a low frequency that can be physically felt, but not heard, by human ears. This frequency was selected because of its low toxicity and few non-specific effects on other aspects of Xenopus development.

Our results clearly implicate two periods of sensitivity to low frequency vibrations. The first period coincides with the first cell cleavage, a developmental window when embryos are sensitive to chemical agents that disrupt the chiral cytoskeleton [31], [34]. Importantly, vibrations from 1 cell to 2 cell alone were not sufficient to alter orientation of the LR axis, but this is similar to what has been seen with most pharmacological treatments that target the cytoskeleton as well [34]. It has been previously proposed that cortical rotation at the 1-cell stage could be the driving force behind the specification of the LR axis, whereby microtubule-driven movements are required to establish both the dorsal-ventral and LR axes [80]. Previous experiments directly addressed the question of whether Spemann's organizer is required for orientation of the LR axis; when UV irradiation – which ablates dorsal-ventral patterning information by blocking cortical rotation – is rescued at the 1-cell stage, the LR axis is typically oriented properly, but in contrast, when dorsal-ventral patterning is restored several cell divisions later, the LR axis is randomized [81]. These experiments indicate the importance of the chiral cytoskeleton in the establishment of LR asymmetry, but challenge the idea that the organizer is sufficient to orient this axis. While the results we have presented could be consistent with vibrations affecting cortical rotation, we limited our analyses to embryos with normal dorsoanterior indexes (Figure 1), suggesting that even subtle changes to the midline were not induced by this treatment.

Our results indicate that aspects of internal dynamics of the embryo, including the movement of high molecular weight dyes throughout the cytoplasm, are affected by low frequency vibration.. Yet our results show that early vibration protocols produce a unique signature compared to reagents that disrupt the cytoskeleton, as well as those that affect other aspects of the ion flux pathway including H^+^ and K^+^ flux, gap junctional communication, and the LR signaling molecule serotonin (Figure 5). Previous studies of the earliest steps in this pathway have utilized reagents that target the chiral cytoskeleton including nocodazole, latrunculin and 2,3-butanedione monoxime (BDM), a drug that inhibits myosin which effectively alters LR patterning only when exposures occur prior to the first cell cleavage [31]. Our results suggest that vibrations during the first stage of development target a structure that is not affected by nocodazole, latrunculin or BDM, even when exposures to those chemicals start during the first cell cleavage. Thus, while the results of our epistasis experiment indicates that the effects of vibration likely affect the same LR pathway as nocodazole (Figure 6A), the physical perturbations appear to also affect some additional aspect of LR patterning, as revealed by the comparison of organ reversal signatures.

Vibrations can disrupt both the cytoskeletal network and the organization of MTOCs in cultured cells [53], [54], and it is plausible that these structures are affected in our embryos. We have previously proposed that the MTOC could be an ancient, highly-conserved mechanism by which asymmetry can be produced in a variety of systems [15], [16] including those observed in single cells [32]. However, vibrated Jurkat cells show growth arrest for a 24-hour period after vibration [54], whereas our embryos develop normally other than problems with laterality. This suggests that if the MTOCs in Xenopus embryos are targeted by low frequency vibrations, the induced alterations in structure would be subtle so as not to disrupt normal cellular processes including cell division. Future studies are needed to explore this hypothesis further.

While the effects of vibration during the first cleavage stage were expected, we were surprised to identify a second period of sensitivity to vibration, a window that overlaps stages 4–7 in the developing Xenopus embryo (Figure 2). These developmental stages correspond to those where the embryo first displays the characteristics of an epithelium including the expression of tight junctions, desmosomes and apical-basal domains [82], although tight junctions are expressed as early as the 4-cell stage (st. 3) [79]. Normal expression of the tight junction protein claudin by the early cleavage stage embryo is an important step in proper LR development (Brizuela et al., 2001). Furthermore, it has been suggested that altered tight junction integrity can prevent the establishment of the electrophysical gradients that are needed for biased localization of the LR signaling molecule serotonin, thereby causing laterality problems [40]. Results from our epithelial permeability assay show that epithelial integrity is disrupted in vibrated embryos (Figure 7), suggesting that vibrations during this period may alter aspects of the ion flux pathway.

**Figure 8:**
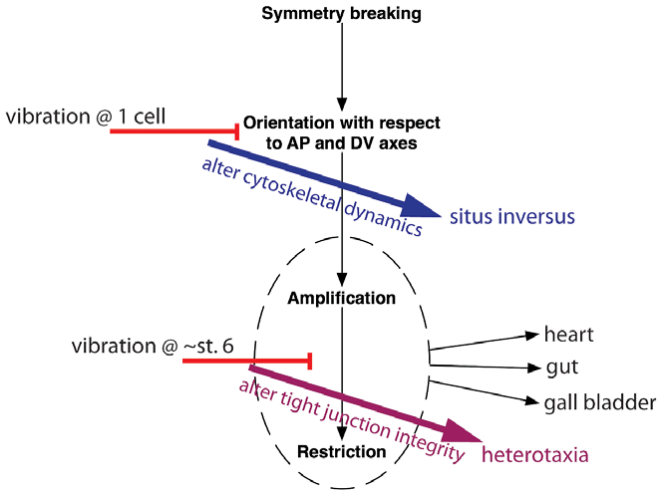
Vibrations affect patterning of the left-right (LR) axis in two distinct ways, producing different phenotypes depending on the timing of treatment. Early events involved in the patterning of the LR axis have been split into three basic steps [1], [42], [94]: breaking of symmetry, orientation of the axes, and amplification/restriction of LR signals. Targeting each of these steps is expected to produce different phenotypes: altered symmetry breaking leads to mirror-image left and right halves, i.e. isomerisms, a LR defect rarely if ever seen in Xenopus but often observed in mutant mice; altered orientation of the axes will produce a mixed population of wildtype and mirror-image individuals (with *situs inversus*) because the LR axis is randomly oriented with respect to the anterior-posterior and dorsal-ventral axes; altered amplification/restriction of LR signals will cause each organ to make independent decisions, thus producing a population of heterotaxic individuals. Here, we show that early vibrations produce large amounts of situs inversus, therefore we propose that the effects of early vibrations on the cytoskeleton are responsible for the second step, proper orientation of the three axes. The effects of vibrations during the second period of sensitivity largely produce heterotaxia, thus we propose that the effects of vibration on tight junction integrity, part of the ion flux pathway, alters the third step in the pathway, the amplification/restriction of LR signals.

In support of this hypothesis, we examined the signature of affected organs in thousands of embryos treated with chemicals and molecular constructs that target different aspects of the ion flux pathway; these reagents produced remarkably similar signatures of affected organs (Figure 5B). While early vibrations, covering the first sensitive period, were significantly different from the embryos where ion flux parameters were affected, vibrations starting at stage 6 were indistinguishable from ion flux (Figure 5C). We thus propose a model explaining how these two periods of sensitivity to vibration could affect different parts of the LR pathway, but both contribute overall to problems with laterality (Figure 8). We observed that vibrations starting at the 1-cell stage disrupt the orientation of the LR axis with respect to the anterior-posterior and dorsal-ventral axes, but maintain high levels of organ concordance. Thus, we propose that by altering an aspect of the chiral cytoskeleton (i.e. the MTOC), early vibrations produce mostly situs inversus. Alternatively, vibrations during the second period of sensitivity altered tight junction integrity, which does not affect alignment of the three axes, but disrupts the amplification and restriction of LR information. This disturbs the concordance of the organs, allowing each organ to make an independent decision about placement, and thus producing heterotaxic embryos.

It is worth noting that while vibration protocols that include the two periods of sensitivity (1–2 cell stage plus stages 4–7) produce more heterotaxia than treatments that target either sensitive period alone (Figure 3 and data not shown), the most penetrant LR phenotypes were observed with prolonged vibrations. For example, vibration from the 1–2 cell stages, plus additional vibration from stage 6 through neurulation produced heterotaxia in over 40% of embryos. This is similar to the incidence of heterotaxia observed when embryos are vibrated undisturbed from 1 cell through neurulation (Figure 2). These results may suggest that there are multiple periods that are sensitive to vibration, and that vibrations across several of these critical periods are needed to maximize the incidence of LR phenotypes.

While the early cytoskeleton and motor proteins have been implicated in LR patterning in a large number of invertebrate species [20], [24], [83], [84] and even plants [25], [26], [27], the only vertebrate model studied in detail is Xenopus [31], [33], [34]. Therefore, this protocol allows for the exploration of mechanistic conservation across additional species including zebrafish and perhaps even the chick [74], [85].

We propose that low frequency vibrations are an alternative to pharmacological treatments that target the cytoskeleton, because unlike exposures to drugs and chemicals, we know exactly when in development the embryo is subjected to treatment. Vibration therefore allows discrete developmental periods to be targeted, and also allows multiple periods to be tested in the same embryos (Figure 3). Additionally, the effects of low frequencies are subtle enough that vibration can be paired with other treatments (Figure 6). In our experience, multiple treatments, each at sub-toxic levels, are typically lethal when combined (Vandenberg & Levin, unpublished data); thus, we have now achieved an experimental system where the effects of altered cytoskeletal dynamics can be paired with treatments targeting other pathways. Of course, vibration protocols are not expected to completely replace pharmacological treatments, but do provide an alternative to these methods when there are concerns about the timing of chemical exposures or when multiple treatments are necessary.

Importantly, knowing exactly when the vibration treatments have ended is not informative about the period of time it takes the embryo to recover from treatment. In cultured cells, the cytoskeleton remains disorganized several hours after vibration, but recovers within 24-hours; cell proliferation resumes sometime after that point [54]. Thus, it is important to address whether the effects of vibration are due to early interruptions of the LR pathway, or whether they could be indicative of effects on other cytoskeletal structures (like cilia) later in development. There are two results that address this issue. First, the striking differences between embryos vibrated from 1 cell through neurulation, compared to embryos vibrated from 2 cell through neurulation, clearly indicates that an extremely early event is affected by this treatment (Figure 2); no differences in the effects on cilia would be expected from these treatments. Second, several treatments that start prior to and span the period of time where ciliary motion and nodal flow occur do not significantly affect the LR axis (i.e. vibration from st. 8 through neurulation; Figure 2). If vibration were affecting the organization or assembly of cilia, these treatments, which fully encompass the developmental periods where cilia appear and flow begins [86], [87], [88], should be effective. Instead, all of our results are consistent with vibration affecting early LR pathways via two mechanisms: disruption of the chiral cytoskeleton and altered epithelial integrity.

In summary, we have identified a new treatment paradigm by which the LR axis of the Xenopus embryo can be disturbed, and our results suggest that the physical perturbations of low frequency vibrations affect the very early cytoskeleton (during the first cleavage stage) and tight junctions. Thus, vibration acts to disrupt two specific steps in the early LR pathway, and ultimately alters patterning of this axis. For the first time, we have identified a treatment that can separately randomize the alignment of the LR axis with the other two axes in addition to disrupting the amplification and restriction of LR information, thus producing different LR phenotypes depending on when the exposures occurred. This information should put scientists in this field one step closer to understanding how the LR axis is consistently oriented and organ concordance is controlled. The question of how symmetry is broken remains a fascinating and provocative question with important relevance to the field of developmental biology as well as medicine, and biophysical tools including the low frequency vibrations explored here offer tremendous power to explore the many puzzles that remain unsolved.

## Materials and Methods

### Animal husbandry

This study was carried out in strict accordance with the recommendations in the Guide for the Care and Use of Laboratory Animals of the National Institutes of Health. The protocol was approved by Tufts University's Institutional Animal Care and Use Committee (#M2008-08). *Xenopus laevis* embryos were collected and fertilized *in vitro* according to standard protocols [89] in 0.1× Modified Marc's Ringers (MMR) pH 7.8+0.1% Gentamycin. *Xenopus* embryos were housed at 18–23°C and staged according to [90].

### Vibration of embryos

4-inch Sony speakers (Model # 1-544-670-11) were connected to a Gwinstek GFG-8216A function generator. Placed on these speakers were embryos (100–200 per treatment) in polystyrene petri dishes (Fisherbrand Catalog # 0875712, diameter = 10 cm, depth = 1.5 cm) containing approximately 40 ml 0.1× MMR. These dishes were vibrated at specific frequencies over various developmental time periods (as indicated in the text). Typically, two speaker systems were positioned on a benchtop near each other, and both were operated at the same time and at the same frequency, set to vibrate at the highest non-lethal amplitude on the sine wave setting for all experiments. When the function generator was set at 7 Hz, the average acceleration of the dish was measured as 0.53 m/sec^2^ using a calibrated Endevco accelerometer (Model 2250K). At 15 Hz, the average acceleration of the dish was 3.96 m/sec^2^. The ambient temperature was always ≤23°C, a temperature that does not affect orientation of the LR axis. Constructive or destructive interference did not appear to occur between the two speakers, and similar results were obtained with single speakers. In each experiment, non-vibrated control dishes were placed in an incubator away from all external sources of vibration.

### EMF treatment

Embryos were placed on 4-inch Sony speakers after removal of the stationary magnet, leaving a speaker that produced electromagnetic fields (EMFs) in the absence of physical vibrations. The speakers were connected to a frequency generator, and all other aspects of the experiment were the same as in the vibration studies.

### Laterality assay

At stage 45, live *Xenopus* embryos were analyzed for position (*situs*) of the heart, stomach and gall bladder according to [76]. Heterotaxia was defined as the reversal in position of one or more organs including situs inversus (reversal of all three organs), which was also considered separately in some experiments. Only embryos with a normal dorsoanterior index (DAI = 5) were scored to prevent confounding of randomization caused by midline defects [91]. Percent heterotaxia was calculated as number of heterotaxic embryos divided by the total number of scorable embryos. A χ^2^ test was used to compare absolute counts of heterotaxic embryos. 10% was set *a priori* as the minimum meaningful difference due to the rate of spontaneous heterotaxia observed in untreated controls (1–3%) and the rates of heterotaxia observed in well-characterized mutants and treatments [17]. For all treatments, experiments were performed in duplicate or triplicate, and the data presented are the sums from those replicates.

### *In situ* hybridization

Whole mount *in situ* hybridization was performed using standard protocols [92]. *In situ* hybridization probes against Xnr-1 (the Xenopus nodal) mRNAs [64] were generated *in vitro* from linearized template using DIG labeling mix (Roche). Embryos vibrated for multiple stages were examined for Xnr-1 localization. A χ^2^ test was used to compare absolute counts of embryos with correct versus incorrect Xnr-1 expression.

### Ternary plots

All embryos with heterotaxia were separated into one of three groups: single organ inversions, double organ inversions, and situs inversus. The percentage of each group was calculated for several vibration periods as well as previously characterized reagents that affect LR orientation (Supplemental Table 2). Each treatment was plotted on a ternary graph using these three measures according to methods described previously [67]. Using the calculations provided by the ternary plot program, https://webscript.princeton.edu/~rburdine/stat/three_categories, when there was no overlap of confidence intervals, results were considered statistically significant. When confidence intervals overlapped, a χ^2^ test was used to calculate statistical significance.

### Epistasis Experiments

For these experiments, embryos were vibrated at 7 Hz, treated with drugs targeting specific LR relevant pathways, or both starting at the 1 cell stage. For drugs concentrations, the following doses were utilized: 2.1 µM lindane [75]; 13 nM nocodazole [34]. All treated embryos remained in the drug solution overnight until stage 12 of development when they were repeatedly washed and then housed in clean 0.1× MMR for the remainder of the experiment. At st. 45, embryos were scored for heterotaxia and the results from each treatment were compared to determine whether there was an additive effect of vibration and drug.

### Tight junction permeability assay

A tight junction assay was performed according to standard protocols [40]. Briefly, control and vibrated embryos were collected at st. 6, cooled to 10°C for 10 min, and then incubated in a 1 mg/ml solution of EZ-Link Sulfo-NHS-LC biotin in 0.1×MMR and 10 mM HEPES (pH = 7.8) at 10°C for 10 min. This biotin molecule has been modified to prevent it from passing through normal tight junctions unless epithelial integrity has been compromised. After labeling, embryos were rinsed twice with cool 0.1×MMR, fixed in MEMFA, processed and embedded in paraffin. 5 µm sections were cut on a Leica M2255 microtome and mounted on glass slides.

The slides were deparaffinized, rehydrated, and blocked with 10% goat serum in 1×PBS for 1 hr at room temperature, then incubated with the fluorescent secondary antibody streptavidin-Alexa 555 at a concentration of 1∶200 in blocking solution. Slides were then washed, mounted with a coverslip, and imaged.

### Intracellular movement assay

Immediately following fertilization, 1 cell embryos were placed in 3% Ficoll and injected with a 1∶1∶2 mixture of Oregon Green (OrG, M_W_ = 10 kDa), Tetramethylrhodamine (TMR, M_W_ = 70 kDa), and RODI water using standard protocols (100 ms pulse time and borosilicate glass needles with a bubble pressure of 60–70 kPa). Injections were uniformly delivered into the middle of the embryo at the animal pole of the embryo. Injected embryos were then either vibrated at 7 Hz for various intervals of time within the first 2 cell divisions or stored in a location with equivalent temperature but no source of external vibration. Following treatment, embryos were fixed in MEMFA, washed, and embedded in agarose according to standard protocols [93]. 100 µm sections were cut using a Leica VT1000S vibratome and imaged in PBS.

### Microscopy

An Olympus BX-61 with a Hamamatsu ORCA AG CCD camera, controlled by MetaMorph software, was used for all fluorescence imaging. For all other experiments, images were collected with a Nikon SMZ1500 dissection microscope with a Retiga 2000R camera and ImageQ software. Photoshop™ was used to orient, scale, and improve clarity of images. Data were neither added nor subtracted; original images are available on request.

## Supporting Information

Figure S1 Embryos were placed on a speaker with the stationary magnet removed, allowing them to be exposed to the same EMFs without any physical vibration. The signal generator was set at 7 Hz. Regardless of the exposure period. EMFs did not affect LR patterning. Siblings collected from the same batches of embryos that were vibrated at 7 Hz had significant amounts of heterotaxia.
(DOC)

Figure S2 Pharmacological (P) and molecular (M) reagents that have been shown to disrupt patterning of the LR axis by targeting the cytoskeleton, H^+^ pumps, K^+^ channels, gap junctional communication (GJC) or serotonin (5HT). (These reagents were previously reported in: [34], [37], [38], [39], [68], [69], [70], [71]).
(DOC)
