# Supplementary material for: Low Frequency Vibrations Disrupt Left-Right Patterning in the Xenopus Embryo
Source: PLoS One. 2011 Aug 3;6(8):e23306. doi: 10.1371/journal.pone.0023306 (PMC3149648; doi:10.1371/journal.pone.0023306)
Supplement: Figure S1 — Embryos were placed on a speaker with the stationary magnet removed, allowing them to be exposed to the same EMFs without any physical vibration. The signal generator was set at 7 Hz. Regardless of the exposure period. EMFs did not affect LR patterning. Siblings collected from the same batches of embryos that were vibrated at 7 Hz had significant amounts of heterotaxia. (DOC) [file pone.0023306.s001.doc]

**Figure S1: No effect of EMFs on laterality defects**

| **Stage of treatment** | **Treatment type** | **% heterotaxia (n)** | **X^2^ value** | **p-value** |
| --- | --- | --- | --- | --- |
| none | Controls (non-vibrated) | 1% (110) | - | - |
| 1 cell – st 19 | EMF | 2% (99) | 0.0 | 0.93 |
| 1 cell – st 8 | EMF | 3% (93) | 0.5 | 0.50 |
| 2 cell – st 19 | EMF | 1% (148) | 0.1 | 0.80 |
| 1 cell – st 19 | Vibrated at 7Hz | 38% (119) | 46.2 | p<<0.001 |
