# Supplementary material for: Low Frequency Vibrations Disrupt Left-Right Patterning in the Xenopus Embryo
Source: PLoS One. 2011 Aug 3;6(8):e23306. doi: 10.1371/journal.pone.0023306 (PMC3149648; doi:10.1371/journal.pone.0023306)
Supplement: Figure S2 — Pharmacological (P) and molecular (M) reagents that have been shown to disrupt patterning of the LR axis by targeting the cytoskeleton, H+ pumps, K+ channels, gap junctional communication (GJC) or serotonin (5HT). (These reagents were previously reported in: [34], [37], [38], [39], [68], [69], [70], [71]). (DOC) [file pone.0023306.s002.doc]

**Figure S2: Pharmacological and molecular reagents reported to affect situs of three organs (heart, gut, and gall bladder)**

| Treatment | Treatment type | Target |
| --- | --- | --- |
| Nocodazole | P | Cytoskeleton |
| Latrunculin | P | Cytoskeleton |
| EHNA | P | Cytoskeleton |
| Dynein antibodies | M | Cytoskeleton |
| As2 |  | Cytoskeleton |
| Rose Bengal lactone | P | Cytoskeleton |
| ME-3407 | P | Cytoskeleton |
| A15 | P | H^+^ pumps |
| A16 | P | H^+^ pumps |
| A6 | P | H^+^ pumps |
| AC45-114 | P | H^+^ pumps |
| Low pH (acidic) MMR | P | H^+^ pumps |
| Allyl isothiocyanate | P | H^+^ pumps |
| bafilomycin | P | H^+^ pumps |
| Concanamycin | P | H^+^ pumps |
| DCCD | P | H^+^ pumps |
| Erythrosin B | P | H^+^ pumps |
| Fusicoccin | P | H^+^ pumps |
| Lansoprazole | P | H^+^ pumps |
| Lobatomide | P | H^+^ pumps |
| Na-ortho-vandate | P | H^+^ pumps |
| NHE3 | M | H^+^ pumps |
| Omeprezole | P | H^+^ pumps |
| Prodigiosin | P | H^+^ pumps |
| SB-242784 | P | H^+^ pumps |
| SCH28080 | P | H^+^ pumps |
| TBT | P | H^+^ pumps |
| YCHE78 | M | H^+^ pumps |
| BaCl | P | K^+^ channels |
| minK | M | K^+^ channels |
| Bir10-ER | M | K^+^ channels |
| Furosemide | P | K^+^ channels |
| Ouabain | P | K^+^ channels |
| Cromakalim | P | K^+^ channels |
| Diazoxide | P | K^+^ channels |
| Valinomycin | P | K^+^ channels |
| Nicorandil | P | K^+^ channels |
| DIDS | P | K^+^ channels |
| PKF-744 | P | K^+^ channels |
| PKF-999 | P | K^+^ channels |
| Glibenclamide | P | K^+^ channels |
| Decanoic acid | P | K^+^ channels |
| PHM | P | K^+^ channels |
| Tolbutamide | P | K^+^ channels |
| Tolazamide | P | K^+^ channels |
| Glipizide | P | K^+^ channels |
| Chlorpropamide | P | K^+^ channels |
| HMR-1098 | P | K^+^ channels |
| U-37883 | P | K^+^ channels |
| Repaglinide | P | K^+^ channels |
| Chromanol 293B | P | K^+^ channels |
| Clofilium | P | K^+^ channels |
| L-768673 | P | K^+^ channels |
| Linopiridine | P | K^+^ channels |
| THB | P | K^+^ channels |
| TAC | P | K^+^ channels |
| Paxilline | P | K^+^ channels |
| Pentylene tetrazol | P | K^+^ channels |
| Lindane | P | GJC |
| Glyc acid | P | GJC |
| Cx32 (ventral injections) | M | GJC |
| H7 (dorsal injections) | M | GJC |
| Heptanol | P | GJC |
| Octanol | P | GJC |
| Canthax | P | GJC |
| Melatonin | P | GJC |
| EM12 | P | GJC |
| Anandemide | P | GJC |
| Oleic acid | P | GJC |
| Mutant Cx32 | M | GJC |
| Dj-Inx11 | M | GJC |
| Dj-Inx12 | M | GJC |
| Dj-Inx5 | M | GJC |
| SB242 | P | 5HT |
| Quercetin | P | 5HT |
| Granestron HCl | P | 5HT |
| Tropesetron | P | 5HT |
| Fluoxetine | P | 5HT |
| Citalopram | P | 5HT |
| Reserpine | P | 5HT |
| TBZOH | P | 5HT |
| Imipramine | P | 5HT |
| Desipramine | P | 5HT |
| Alaproclate | P | 5HT |
| 8-hydroxy-DPAT | P | 5HT |
| RS-67506 | P | 5HT |
| Chlorophenylbiguanine | P | 5HT |
| RS-67333 | P | 5HT |
| Methylsergide | P | 5HT |
| Methiothepin | P | 5HT |
| Metergoline | P | 5HT |
| Cinanserine | P | 5HT |
| SDZ-205,930 | P | 5HT |
| LY-278,584 | P | 5HT |
| ICS-205,930 | P | 5HT |
| GR113,808 | P | 5HT |
| MDL 74444 | P | 5HT |
| Iproniazid | P | 5HT |
| Harmine | P | 5HT |
| Pargyllene | P | 5HT |
| Clorgyline | P | 5HT |
| Phenelzine | P | 5HT |
| Furazolidone | P | 5HT |
| Serotonin (injected) | P | 5HT |
| Parthenolide | P | 5HT |
